# X-Sens Inertial Sensor Technology-Based Rehabilitation on a Patient With Posterior Cruciate Ligament Avulsion Fracture and Shaft of Femur Fracture: A Case Report

**DOI:** 10.7759/cureus.55217

**Published:** 2024-02-29

**Authors:** Mahek Mohani, HV Sharath, Tanvi Varma

**Affiliations:** 1 Department of Paediatric Physiotherapy, Ravi Nair Physiotherapy College, Datta Meghe Institute of Higher Education and Research (Deemed to be University), Wardha, IND; 2 Department of Cardiovascular and Respiratory Physiotherapy, Ravi Nair Physiotherapy College, Datta Meghe Institute of Higher Education and Research (Deemed to be University), Wardha, IND

**Keywords:** midshaft femur fracture, gait analysis, gait, physiotherapy, pcl avulsion fracture

## Abstract

The posterior cruciate ligament (PCL), one of the key ligaments in the knee, serves to prevent backward movement of the tibia relative to the femur. A simultaneous occurrence of a PCL avulsion fracture and a femur shaft fracture in a pediatric patient suggests a complex orthopedic injury resulting from significant trauma to the knee and thigh area. This study describes the rehabilitation process of a 12-year-old female involved in a road traffic accident, who suffered both a midshaft femur fracture and a PCL avulsion fracture. Following surgical procedures, the patient underwent a comprehensive physiotherapy regimen utilizing X-Sens inertial sensor technology. The rehabilitation plan comprised multiple stages targeting pain alleviation, muscle strengthening, flexibility exercises, gait retraining, and balance improvement. Various interventions including contrast baths, cryotherapy, patellar mobilization, isotonic and resistance exercises, and progressive gait training were integrated across different phases of the rehabilitation program. Over subsequent follow-up periods, the patient demonstrated significant enhancements in pain management, range of motion, muscle strength, functional capabilities, and gait metrics. This case report underscores the efficacy of a systematic physiotherapy strategy incorporating advanced technology in the successful recovery from intricate lower limb fractures, underscoring the importance of prompt intervention and multidisciplinary collaboration for optimal patient outcomes.

## Introduction

Bearing the weight of the entire body, the femur is the most powerful and longest bone in the body. Being the heaviest tubular bone in the body, it can break with tremendous energy force, such as in a car accident. Ten midshaft femur fractures per 100,000 person-years are reported to occur each year [1]. A bimodal distribution characterizes the incidence of femoral diaphyseal fractures, with a peak in young people and the elderly. This is likely due to high-energy mechanisms in younger individuals and low-energy falls in older individuals with lower bone density. The main weight-bearing bone in the lower extremities is the femur. Both with and without the assistance of surgery, the degree of quadriceps femoris muscle weakness is frequently a cause for worry in cases of femur fractures [2].

They are often treated surgically, and after surgery, immediate physiotherapy care is needed. Deep venous thrombosis (DVT), atrophy, discomfort, stiffness, and decreased muscle strength are among the postoperative consequences. Immobilization is the main cause of these problems [3]. High-risk side effects from a femur fracture include bleeding, fat embolism, and infection. Additionally, extended morbidity with shortening, misalignment, and deep venous thrombosis (DVT) can result from improper management of a femur fracture [4,5].

Early mobility after a femur shaft fracture reduces the incidence of quadriceps and hamstring atrophy, as well as hip and knee stiffness. Achieving instant weight-bearing and making advancements in gait training should be the main priorities [6]. Research has indicated a correlation between the assessed displacement of the fracture and the weakness of the quadriceps femoris muscle, suggesting that the discovered strength deficiency may possibly be the consequence of muscular damage from the injury. Following surgical treatment of a midshaft femur fracture, immediate weight-bearing combined with early strengthening exercises may lead to the early cure of impairments and functional limits, as well as decreased disability.

The strongest ligament in the knee, the posterior cruciate ligament (PCL), is essential for preserving knee stability. It prevents the tibia from sliding backward with respect to the femur by extending obliquely backward and downward from the medial femoral condyle. The posteromedial and anterolateral bundles make up the PCL, which adds to its sturdy construction. Its principal role is to impede the posterior displacement of the tibia, especially when knee flexion and extension, rotation, and dynamic balancing exercises are involved. Particularly in frontal aggression in the flexed knee posture or hyperextension, the PCL is vulnerable to injury. The PCL is involved in 4%-38% of knee injuries observed in emergency rooms, indicating the clinical importance of this ligament in preserving knee joint integrity [7]. Avulsion fractures of the tibial insertion caused by the posterior cruciate ligament (PCL) are extremely uncommon in pediatric patients. For an accurate diagnosis and course of treatment, radiological investigations are essential in addition to a thorough clinical evaluation. Its managerial style can be operational or conservative [8,9].

After a posterior cruciate ligament (PCL) injury, long-term consequences may include ongoing instability, reduced activity, and degenerative changes. To address a displaced bony avulsion of the PCL surgically and prevent malunion or nonunion, restoring knee stability is crucial [10,11]. Gait analysis reveals that the tibia experiences more compression than the femur during walking, primarily due to the activation of plantar flexor muscles in the late stance phase, especially during push-off. In terms of bending moments, the femur exhibits higher mean values in the frontal plane compared to the tibia, with decreasing values toward the distal end of each bone. In the sagittal plane, the femur's mean bending moments remain modest, reaching high values at the proximal end of the tibial shaft and decreasing distally [12,13].

One such advancement is the integration of inertial sensor technology, exemplified by X-Sens, into rehabilitation protocols. These sensors enable precise and objective monitoring of joint movements, muscle activation patterns, and functional performance during exercises. By providing real-time feedback and quantifiable data, X-Sens technology enhances the precision and individualization of rehabilitation programs [14]. This case report outlines a comprehensive physiotherapy intervention utilizing X-Sens inertial sensor technology in the rehabilitation of a patient diagnosed with concurrent PCL avulsion fracture and shaft of femur fracture. The report details the patient's presentation, rehabilitation process, and outcomes and highlights the role of X-Sens technology in optimizing rehabilitation strategies and patient outcomes. Through this case report, we aim to underscore the potential benefits and efficacy of incorporating inertial sensor technology into physiotherapy practice for complex orthopedic injuries [15].

Following surgery, we recommend weight-bearing on the flat of the foot and permit a passive range of motion right away, under the guidance of physical therapy. Four types of postoperative interventions are used: balance training, aquatic therapy, strengthening exercises, and clinical environments. The best protocols for outpatient physical therapy should involve strengthening and intense functional exercises delivered through programs that are either land-based or aquatic, with the intensity of the exercises being increased in accordance with the patient's progress. The objective of the study is to assess the effectiveness of X-Sens inertial sensor technology in rehabilitating a patient with both posterior cruciate ligament avulsion fracture and shaft of femur fracture [16].

## Case presentation

Patient information

We present a case of a 12-year-old female who met with a road traffic accident on 17th July 2023 while getting out of an auto-rickshaw where a bike suddenly hit her, and so she was brought to the hospital casualty for further management. X-ray was performed, which suggested a midshaft femur fracture and posterior cruciate ligament avulsion fracture of the right side. The patient was operated on for the same on 19th July 2023 and then, after the operation, was shifted to the female orthopedic ward (unit 2, bed number 12). The patient complained of pain at the suture site over the right thigh, which was sudden in onset, progressive in nature, high in intensity, sharp shooting, and aggravated on slight movement, and does not even relieve on rest. For this, physiotherapy was advised. The patient belongs to the upper lower class according to the modified Kuppuswamy scale. The timeline of events is mentioned in Table 1.

**Table 1:** Timeline of events

Events	Date
Date of admission and date of surgery	19th July 2023
Date of physiotherapy rehabilitation	22nd July 2024
Date of discharge	25th August 2023

Clinical findings

Before the commencement of the examination, patient informed consent was obtained. The patient was conscious, cooperative, and well-oriented to time, place, and person. On observation, the build of the patient was ectomorphic, and no edema or muscle wasting was present. On palpation, tenderness grade 3 was present over the medial aspect of the right thigh, lateral aspect of the distal thigh, and around the popliteal fossa of the right lower extremity. The skin type was found to be dry, and swelling was present over the right thigh and medial aspect of the right thigh. Approximately 4 cm incision was taken over the popliteal fossa, approximately 2 cm incision was taken over the medial aspect of the thigh, and approximately 2 cm incision was taken over the lateral aspect of the distal thigh. Deformity was present over the right thigh. On rating pain using the Numerical Pain Rating Scale (NPRS), the patient rated 1/10 on rest and 8/10 on movement. The motor examination of the patient and manual muscle testing are mentioned in Table 2 and Table 3, respectively.

**Table 2:** Range of motion (motor examination) AROM: active range of motion, PROM: passive range of motion, Rt: right, Lt: left

Joint	Pre-rehab		Follow-up (1-3 months)		Follow-up (3-5 months)		Follow-up (5-8 months)	
	AROM (Rt)	PROM (Rt)	AROM (Rt)	PROM (Rt)	AROM (Rt)	PROM (Rt)	AROM (Rt)	PROM (Rt)
Hip flexion	10°	20°	70°	80°	90°	100°	110°	120°
Hip extension	5°	8°	10°	15°	18°	22°	25°	30°
Hip abduction	10°	14°	18°	24°	28°	30°	40°	45°
Hip adduction	10°	12°	15°	18°	20°	24°	26°	30°
Medial rotation	5°	10°	10°	20°	25°	30°	40°	45°
Lateral rotation	5°	10°	10°	15°	20°	30°	40°	45°
Knee flexion	30°	40°	45°	70°	100°	110°	120°	135°
Ankle dorsiflexion	5°	8°	10°	12°	15°	18°	18°	20°
Ankle plantarflexion	10°	16°	20°	25°	30°	38°	45°	45°
Inversion	5°	12°	15°	20°	25°	28°	30°	35°
Eversion	5°	8°	10°	12°	14°	16°	18°	20°

**Table 3:** Manual muscle testing

Muscles	Pre-rehab	Follow-up (1-3 months)	Follow-up (3-5 months)	Follow-up (5-8 months)
	Right	Right	Right	Right
Hip flexors	1/5	3/5	4/5	5/5
Hip extensors	1/5	3/5	4/5	5/5
Hip abductors	2/5	3/5	4/5	5/5
Hip adductors	2/5	3/5	4/5	5/5
Medial rotators	2/5	3/5	4/5	5/5
Lateral rotators	2/5	3/5	4/5	5/5
Knee flexors	2/5	3/5	4/5	5/5
Ankle dorsiflexors	3/5	3/5	4/5	5/5
Ankle plantar flexors	3/5	3/5	4/5	5/5
Ankle invertors	3/5	3/5	4/5	5/5
Ankle evertors	3/5	3/5	4/5	5/5

Investigation details

**Figure 1:**
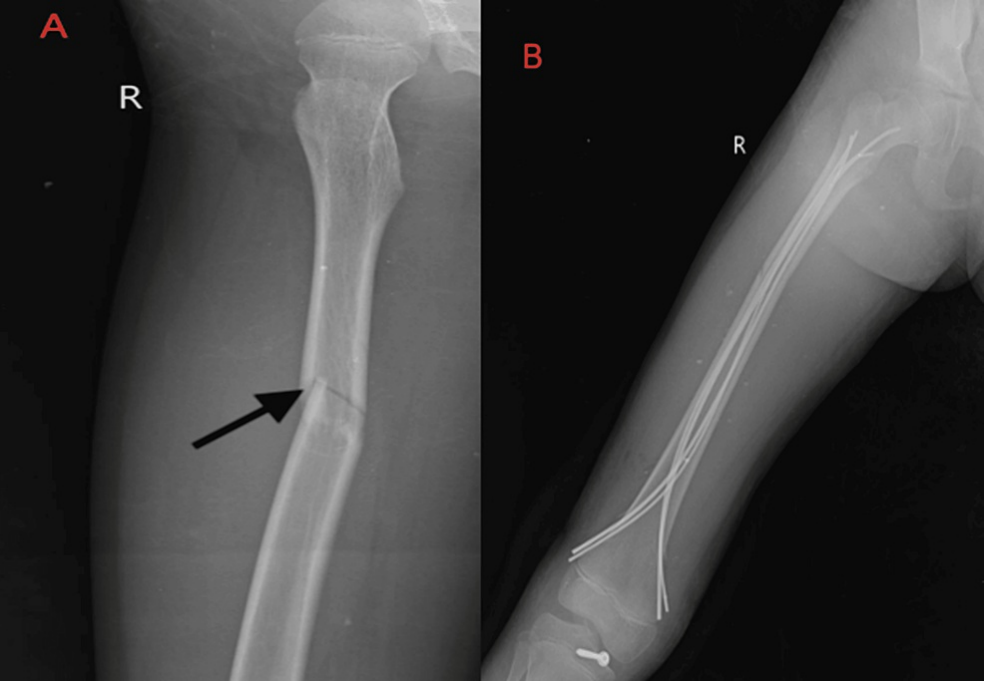
Preoperative (A) and postoperative (B) investigations A: Midshaft femur fracture and posterior cruciate ligament avulsion fracture of the right side. B: Open reduction and internal fixation.

The investigation highlights the implementation of X-Sens inertial sensor technology in tracking and enhancing the rehabilitation outcomes of a patient undergoing treatment for coexisting posterior cruciate ligament avulsion fracture and shaft of femur fracture. Detailed investigation (X-ray) is seen in Figure 1A, and the postoperative investigation report is mentioned in Figure 1B.

Surgery details

In the first surgical procedure, open reduction and internal fixation was performed using Trochanteric Entry Nail System (TENS) nailing for a midshaft femur fracture on the right side. The patient was positioned supine on the operating table under strict aseptic precautions, and the fracture site was visualized using C-arm guidance. A lateral approach was adopted from the distal thigh, involving a 2 cm incision over the lateral aspect. Soft tissues were retracted, and the tensor fascia lata was mobilized to gain access. Using a bone awl, two 3.5 mm TENS nails were inserted laterally from the distal to the proximal direction, reaching up to the greater trochanter. Subsequently, a medial approach was taken with a 2 cm incision over the medial aspect of the thigh. Soft tissues were dissected, and the vastus medialis was retracted for entry. Two additional 3.5 mm TENS nails were inserted medially from the distal to the proximal direction, extending up to the femoral neck. Final fixation was confirmed under C-arm guidance, followed by closure using Ethilon 2-0 and sterile dressing. The patient was then transferred to the recovery room.

In the second surgical procedure, open reduction and internal fixation was performed with a cannulated cancellous (CC) screw for the posterior cruciate ligament (PCL) avulsion fracture on the right side. The patient was placed in a supine position on the operating table with aseptic precautions in place. A 4 cm incision was made over the popliteal fossa, and soft tissues were dissected with retraction of the medial head of the gastrocnemius. The PCL avulsion fragment was visualized and fixed using a CC screw and washer under C-arm guidance. Closure was achieved with Ethilon 2-0, and a sterile dressing was applied before the patient was referred to the recovery room.

Intervention

**Figure 2:**
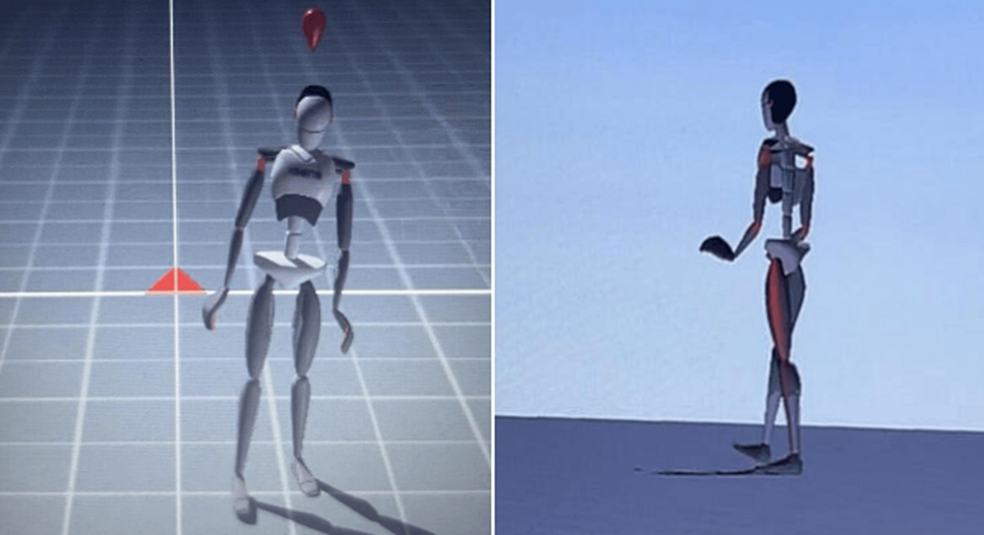
X-Sens inertial sensor technology-based gait training (using avatar)

Physiotherapy intervention incorporated a combination of manual therapy, therapeutic exercises, neuromuscular re-education, and gait training. The rehabilitation program was personalized based on the patient's functional deficits and rehabilitation goals. Central to the intervention was the integration of X-Sens inertial sensor technology for real-time monitoring(Figure 2) of joint angles, movement patterns, and muscle activation during exercises (Figure 2).

The X-Sens inertial sensor technology-based rehabilitation enabled precise quantification of progress and adjustments to the rehabilitation protocol as needed as mentioned in Table 4. Over the course of eight months, the patient exhibited significant improvements in range of motion, muscle strength, and functional mobility. Objective data collected through X-Sens technology demonstrated progressive gains in joint stability and movement coordination throughout the rehabilitation process. By the end of the intervention, the patient achieved a near-normal gait pattern and functional independence in activities of daily living.

**Table 4:** Physiotherapeutic intervention using X-Sens inertial sensor technology feedback Source: [17]

Phase 1	Phase 2	Phase 3	Phase 4
Intervention: Contrast bath, goals: to decrease the intensity of pain and to facilitate the blood flow to the affected area, intensity: 10 minutes before and after the treatment	Intervention: Contrast bath, goals: to decrease the intensity of pain and to facilitate the blood flow to the affected area, intensity: 10 minutes before and after the treatment	Intervention: Cryotherapy, goals: to decrease the intensity of pain and to facilitate the blood flow to the affected area, intensity: 10 minutes before and after the treatment	Intervention: Contrast bath, goals: to decrease the intensity of pain and to facilitate the blood flow to the affected area, intensity: 10 minutes before and after the treatment
Intervention: Cryotherapy, goal: to reduce edema, intensity: 10 minutes before and after treatment	Intervention: Patellar mobilization by providing superior, inferior, medial, and lateral glide, goal: to improve patellar mobility, intensity: 6 repetitions × 1 set Maitland mobilization	Intervention: Patellar mobilization by providing superior, inferior, medial, and lateral glide, goal: to improve patellar mobility, intensity: 6 repetitions × 1 set Maitland mobilization	Intervention: Patellar mobilization by providing superior, inferior, medial, and lateral glide, goal: to improve patellar mobility, intensity: 6 repetitions × 1 set Maitland mobilization
Intervention: Isometric exercises of the quadriceps, gluteus maximus, minimus, and medius, and hamstrings, goals: to improve the strength of the quadriceps, hamstrings, gluteus maximus, minimus, and medius, intensity: 10 repetitions × 1 set	Intervention: Begin resisted exercises with minimum weight for strengthening the quadriceps, gluteus minimus, maximus, and medius, and hamstrings, goals: to improve the strength of the quadriceps, hamstrings, and gluteus maximus, minimus, and medius, intensity: 10 repetitions × 2 sets	Intervention: Progressive resisted exercises to strengthen the hamstrings, quadriceps, and gluteus maximus, minimus, and medius, goals: to improve the strength of the quadriceps, hamstrings, and gluteus maximus, minimus, and medius, intensity: 10 repetitions × 3 sets with weight cuffs	Intervention: Progressive resisted exercises to strengthen the hamstrings, quadriceps, and gluteus maximus, medius, and minimus, goals: to improve the strength of the quadriceps, hamstrings, and gluteus maximus, minimus, and medius, intensity: 10 repetitions × 3 sets with Therabands
Intervention: Isotonic ankle exercises such as ankle toe movements and mild stretching exercises are given to lower limb muscles, goals: to improve the strength of the gastrocnemius and prevent plantar flexor contracture formation and shortening or tightness of the lower limb, intensity: 10 repetitions × 2 sets	Intervention: Deep friction, firm massage, skin and muscle rolling, isotonic ankle exercises such as ankle toe movements and mild stretching, exercises are given to lower limb muscles, goals: to improve scar mobility and the strength of the gastrocnemius and prevent plantar flexor contracture formation and shortening or tightness of lower limb muscles, intensity: 10 repetitions × 2 sets	Intervention: Full weight-bearing without the use of assisted devices, parallel bar walking in front of a posture mirror, and stair climbing 2 times, goal: gait training, intensity: 2-4 rounds	Intervention: Backward walking, tandem walking, goal: gait training, intensity: 2 times
Intervention: Two-point and then three-point gait with crutches is taught to the patient, goal: gait training, intensity: 1 round	Intervention: Partial weight-bearing using crutches or walker, goal: gait training, intensity: 1 round	Intervention: Wobble board balancing, Frenkel exercises, goal: balance training, intensity: 10 repetitions × 2 sets	

Outcome measures 

Follow-up assessments at three, six, and eight months (Table 5) post-intervention revealed sustained improvements with no reported complications.

**Table 5:** Outcome measure FIM: Functional Independence Score, KOOS: Knee Injury and Osteoarthritis Outcome Score, PEDI-IKDC: Pediatric International Knee Documentation Committee Subjective Knee Evaluation Form, NPRS: Numerical Pain Rating Scale

Outcome measures	Pre-rehab	Follow-up (1-3 months)	Follow-up (3-5 months)	Follow-up (5-8 months)
FIM	1	3	5	7
KOOS Child	10	30	50	80
PEDI-IKDC	20	40	70	90
Wong-Baker Scale	10	6	4	2
NPRS	8	6	4	1

## Discussion

The case report presents a comprehensive rehabilitation program for a 12-year-old female with concurrent midshaft femur fracture and PCL avulsion fracture following a road traffic accident. The rehabilitation protocol incorporated various phases targeting pain management, muscle strengthening, range of motion exercises, gait training, and balance enhancement. Innovative technology such as X-Sens inertial sensor technology was utilized throughout the rehabilitation process, showcasing its effectiveness in achieving significant improvements in pain levels, range of motion, muscle strength, functional outcomes, and gait parameters. The introduction provides a detailed overview of midshaft femur fractures and PCL injuries, emphasizing their clinical significance, treatment modalities, and potential complications. It discusses the importance of early mobility and rehabilitation interventions in improving outcomes and preventing long-term complications such as muscle weakness, joint stiffness, and instability. The case presentation offers a thorough examination of the patient's clinical findings, surgical interventions, and rehabilitation timeline. It includes detailed assessments of range of motion, manual muscle testing, and surgical procedures, providing valuable insights into the patient's condition and progress over time [18].

X-Sens inertial sensors provide a means for objective assessment of patient movement and progress throughout the rehabilitation process. By capturing motion data in real time, clinicians can track improvements in range of motion, joint stability, and functional movement patterns. This objective data offers valuable insights into the effectiveness of the rehabilitation program and allows for adjustments to be made as needed. One of the notable advantages of X-Sens technology is its ability to facilitate personalized rehabilitation protocols. By analyzing the specific movement patterns and biomechanics of the patient, clinicians can tailor the rehabilitation program to address individual deficits and challenges. This personalized approach is essential for optimizing outcomes and minimizing the risk of complications or re-injury.

The real-time feedback provided by X-Sens inertial sensors enables both patients and clinicians to monitor progress during rehabilitation sessions. Immediate feedback on movement quality, symmetry, and biomechanics allows for adjustments to be made in real time, promoting more efficient and effective rehabilitation outcomes. Additionally, continuous monitoring of patient movement outside of clinical settings can help ensure compliance with prescribed exercises and activity modifications. X-Sens technology can complement traditional rehabilitation techniques by providing additional insights into movement mechanics and compensatory patterns. Integrating inertial sensor data with other assessment tools, such as gait analysis or manual muscle testing, allows for a more comprehensive understanding of the patient's condition and progress. This integrated approach enhances the clinician's ability to design targeted interventions and optimize rehabilitation outcomes.

While X-Sens inertial sensor technology offers many benefits for rehabilitation, it is not without its challenges and limitations. Factors such as cost, accessibility, and technical expertise required for implementation may limit its widespread adoption. Additionally, the accuracy and reliability of motion capture data can be influenced by factors such as sensor placement, calibration, and environmental conditions. Clinicians must be aware of these limitations and use inertial sensor data in conjunction with other clinical assessments to make informed decisions about patient care [19,20].

The physiotherapy intervention section outlines a structured rehabilitation program consisting of various modalities such as contrast baths, cryotherapy, patellar mobilization, isotonic and resisted exercises, and gait training. Each intervention is tailored to address specific goals related to pain management, muscle strength, joint mobility, and functional recovery. The outcome measures highlight the patient's progress throughout the rehabilitation process, including improvements in functional independence, pain levels, and quality of life. The discussion section critically analyzes the effectiveness of the rehabilitation program and highlights the importance of multidisciplinary care and innovative technology in optimizing patient outcomes. In conclusion, the case report highlights the potential of X-Sens inertial sensor technology as a valuable tool in the rehabilitation of patients with complex musculoskeletal injuries. By providing objective assessment, personalized rehabilitation protocols, real-time feedback, and integration with traditional techniques, X-Sens technology can help optimize outcomes and improve patient care in rehabilitation settings. However, it is essential for clinicians to recognize the challenges and limitations associated with this technology and use it judiciously as part of a comprehensive rehabilitation approach. Further research and clinical experience are needed to fully understand the role of inertial sensor technology in rehabilitation and its impact on patient outcomes.

## Conclusions

In conclusion, this case report demonstrates the successful rehabilitation of a pediatric patient with concurrent midshaft femur fracture and posterior cruciate ligament (PCL) avulsion fracture using a structured physiotherapy program incorporating X-Sens inertial sensor technology. The multidisciplinary approach, including surgical interventions followed by early and comprehensive physiotherapy care, led to significant improvements in pain levels, range of motion, muscle strength, functional outcomes, and gait parameters. This case report underscores the importance of early intervention, multidisciplinary collaboration, and the integration of technology in the rehabilitation of complex lower limb fractures. It highlights the efficacy of tailored physiotherapy programs in achieving optimal patient outcomes and emphasizes the need for individualized care in pediatric orthopedic rehabilitation. By documenting the clinical course, interventions, and outcomes of this case, we aim to contribute to the body of evidence supporting effective rehabilitation strategies for similar complex orthopedic injuries in pediatric patients. Further research and clinical studies are warranted to validate the findings of this case report and explore additional innovative technologies and rehabilitation approaches in pediatric orthopedic rehabilitation.
